# Effect of body-related information on food attentional bias in women with body weight dissatisfaction

**DOI:** 10.1038/s41598-023-43455-6

**Published:** 2023-10-04

**Authors:** Pei Xie, Han-Bin Sang, Chao-Zheng Huang, Ai-Bao Zhou

**Affiliations:** 1 College of Psychology, Sichuan Normal University, Chengdu, 610066 China; 2 Key Laboratory of Behavioral and Mental Health, Gansu Province, China; 3 Department of Psychology, School of Psychology, Northwest Normal University, Lanzhou, 730070 China; 4 Key Laboratory of Child Cognition & Behavior Development of Hainan, Haikou, 570100 China; 5 School of Teacher Education, Qiongtai Normal University, Haikou, 570100 China; 6 Gansu University of Political Science and Law, Lanzhou, China

**Keywords:** Human behaviour, Weight management, Nutrition

## Abstract

Women with body weight dissatisfaction (BWD) have long-term negative assessments of their body weight, which are often associated with poor eating behavior. In this study, we investigated the effect of body-related information on the food cue processing and attention of women with BWD. Sixty-eight women were recruited and assigned to either a BWD (NPSS-F > 2) (*n* = 32) or a no body weight dissatisfaction (NBWD) group (NPSS-F < 1) (*n* = 36). We measured attentional bias to food cues (high- and low-calorie) with a food probe task after exposure to body-related information and recorded eye tracking data. Body-related images were presented prior to a pair of stimulus images (food–neutral or neutral–neutral). Body-related information and food type were repeated measure factors in our study. Our results showed that the first fixation duration bias for high-calorie foods was significantly longer than for low-calorie foods after exposure to overweight cues in the BWD group. Compared with the NBWD group, the BWD group showed longer first fixation duration bias for high-calorie foods after exposure to overweight cues. The direction for high-calorie foods was significantly more often than that for low-calorie foods in the BWD group after exposure to body-related information. Our findings suggest that compared to women with NBWD, women with BWD may be more susceptible to body-related information, resulting in increased attention to high-calorie foods.

## Introduction

Body weight dissatisfaction (BWD) is a condition in which an individual has a negative body image because they perceive themselves as overweight or underweight^1, 2^. Dumas and Desroches suggested that the impact of social media on body image outcomes is mostly detrimental in adolescent girls and women, and is highly associated with exposure to idealized social media images^3^. Tiggemann suggested that women’s body dissatisfaction was remarkably stable across their life span, especially weight dissatisfaction^4^. BWD is not only a risk factor but is also a substantial symptom for diet-related diseases (e.g., bulimia nervosa and anorexia nervosa)^2^. Although studies have shown that BWD can positively predict subsequent food addiction^5, 6^, the cognitive processes related to eating behavior in women with BWD remain unclear. Food attention as an objective index of motivational state can clarify the cognitive mechanisms and role of correlative influencing factors of eating behavior in women with BWD^7, 8^.

Food attentional bias is the tendency to focus on food rather than neutral information^9^. Cognitive processes—such as attention—underpin everyday eating behaviors^9–11^, and food attentional bias can predict subsequent food intake and the type of intake^12^. Wells and Matthews^13^ theorized that within a processing framework (the self-regulatory executive function) attentional bias is related to personality traits. For example, the attentional bias of individuals with high anxiety can change with threatening information^14, 15^. Studies have found a reliable link between exposure to thin, idealized fashion media and body dissatisfaction^16^, as women critically compare their bodies to the slender figures presented in the media, leading to increased negative mood and body dissatisfaction^17^—thus, body-related information may be a threat stimulus for women with BWD^18^. Dreier et al.^19^ found that individuals with disordered eating exhibit greater attentional biases toward body stimuli compared to healthy controls, and attentional bias could reflect an approach motivation (the will to engage with a stimulus) or an avoidance motivation (the will to cease engagement with a stimulus)^12^. The body also represents an anxiety-provoking construct. To avoid dealing with this adverse stimulus, individuals may exhibit attentional bias to other immediate stimuli of environments—most likely hedonic stimuli (e.g., food)—to escape negative self-awareness^20, 21^. According to the theory of food reward^20^, delicious food can provide a taste reward—which is essentially a hedonic stimulus. Although healthcare professionals and the media advocate healthy diets, many individuals intuitively believe that unhealthy is tasty^21^ albeit high in calories^22^. This intuitive belief may operate at an implicit level, which may override the motivation of healthy diets^21^.

Based on this cognitive theory, Williamson^23^ suggested that overfocusing on body size or shape can result in a body self-schema that is readily activated by external stimuli^24^. Women with BWD can easily be influenced by slender, attractive images in the body-related information surrounded by mass media^25^. Fuller-Tyszkiewicz et al.^26^ used a baseline survey and a modified dot probe task to explore the effect of experiences of body dissatisfaction in quotidian life by examining attentional bias toward body shape and weight information. To measure attentional biases, Fuller-Tyszkiewicz et al. presented paired body images: an underweight body was paired with a neutral (average weight) body; an overweight body was paired with a neutral body, and the images for 100 ms or 500 ms. Subsequently, a dot appeared in the location of one of the two body images. They instructed participants to use the keypad to identify the location of the dot (right or left) as quickly as possible. The results showed that appearance-related information led to short-term attentional biases, suggesting that this could be a vulnerability factor for the prolonged persistence of body dissatisfaction experiences. Similarly, Gao et al.^27^ posited that when women with body dissatisfaction are given body-related information, they often engage in social comparison and activate self-references, which may increase cognitive biases for their body weight or body shape and reinforce a maladaptive body schema. According to the cognitive behavioral model, certain types of stimuli (such as body-related information) easily activate cognitive biases in individuals with a highly developed body schema and then direct their attention to food cues^24^.

In the contemporary media-intensive environment, body-related information is ubiquitous (e.g., images of underweight models in magazines or in social media). For women already dissatisfied with their body weight, this information represents a type of weight-related teasing, and may induce a series of unhealthy eating behaviors^28^. Yet, scant scholarly research on the effect of body-related information on eating behavior exists—particularly from cognitive perspectives—and the study of attentional bias of food cues can facilitate the exploration of this process^29^. Dot probe and visual search tasks, and spatial cue paradigms are now widely used in attentional bias studies^26, 30^. These paradigms, however, cannot examine dynamic attentional processes, and thus require additional scientific methods. In this study, we evaluated the strength of the association in a meta-analysis of a sample of 68 Chinese women, deriving correlation coefficients between subjective craving and attentional bias indices^31^. Our results suggested that attentional bias was corrected with subjective craving—and eye tracking is the most sensitive indicator for measuring attentional bias. It not only clearly records the dynamic changes of attention within a certain range over time, but also identifies the components of these attention processes. This supports the extant research on the reliability of attentional bias scores from the food dot probe task, that has found that eye tracking can produce more reliable food attentional bias results^32^.

This study investigated the effect of body-related information on food-related attentional bias in women with BWD. Media images of overweight and underweight individuals lead them to develop downward or upward comparisons, and then change their self-evaluation^33^. In the present experiment, the role of body-related information images present as a stimulus to activate body self-schema of participants, which effectively represent a priming process. The process of priming is usually used in emotion task. For example, Lihua and Caibin^34^ used affective pictures to activate participants' different emotions. Affective pictures were present after a black central fixation cross for 500 ms, followed by an affective picture in the center of screen for 1000 ms as emotions priming, followed by a dot probe task. The results showed that different affective picture can effectively activate different emotions. Gao et al.^27^ found that women with body dissatisfaction are much more affected by overweight or underweight body images when compared with controls. By using free view tasks combined with eye movement tracking to investigated attentional biases to body images among women with body dissatisfaction, they found that women with body dissatisfaction showed sustained attention bias on underweight and overweight body images during both the early automatic and the late strategic processing stages. In this study, we investigated the effect of body-related information on food-related attentional bias of women with BWD compared to women without BWD. Following a literature review^16, 18, 27^, we hypothesized body-related information could affect food attentional bias of women with BWD, and exploratory hypothesized that—compared to women without BWD—women with BWD have more attentional bias toward high-calorie foods after exposure to body-related information.

## Methods

### Participants

Three hundred female college students from Northwest Normal University in Lanzhou, Gansu Province of the People's Republic of China (China) were recruited to complete the Negative Physical Self Scale-Fatness subscale (NPSS-F) through an online survey^1^. Students who met the criteria were invited to participate by telephone or by email. The exclusion criteria were as follows: (a) psychotic disorder, self-reported history of psychological or psychiatric diagnosis; (b) self-reported current dieting or experience of any eating problems (diet, partial eclipse, or erratic); (c) eating disorders, self-reported history of anorexia nervosa or bulimia nervosa diagnosis, or (d) substance abuse, self-reported smokers, self-reported alcohol drinkers, self-reported current medication.

Following Gao et al.^35^, participants whose average score on the NPSS-F was higher than 2 were classified into the BWD group, and those with a score lower than 1 were classified into the NBWD group. BWD group and NBWD group were matched on demographic characteristics and hunger (see Table 1). Participants must have normal or corrected-to-normal vision.

**Table 1:** Basic characteristics of participants.

	BWD (*M* ± *SD*)	NBWD (*M* ± *SD*)	*t*
*n*	32	36	
Age	21.72 ± 3.04	22.64 ± 2.63	1.33
BMI (kg/m^2^)	23.70 ± 2.77	19.42 ± 1.91	7.47***
Hunger	78.59 ± 6.63	78.47 ± 8.00	0.07
NPSS-F	2.36 ± 0.34	0.69 ± 0.35	19.42***

Three asterisks (***) indicate *p* < .001.

*M* mean, *SD* standard deviation.

An a priori power analysis was conducted using G*Power version 3.1.9.2^36^ to determine the minimum sample size required to test the study hypothesis. Results indicated the required sample size to achieve 80% power for detecting a medium effect (*f* = 0.2), at a significance criterion of *α* = 0.05, was *N* = 42 for the interaction of 2 × 3 repeated measures in the analysis and interpretation of ANOVA. To avoid excluded eye movement data leading to insufficient sample size, the sample size in this study is more than 42.

According to the power analysis described in the methods of similar studies^27, 35, 37^, the sample size ranges from 40 to 68. Therefore, we recruited a total of 68 participants in this study. Table 1 shows the general characteristics of participants. All participants received 30 RMB after they finished the whole procedure.

### Ethics statement

This study protocol was approved by the Ethics Board of Northwest Normal University (ERB No. 2019345, dated on 05/12/2019). Informed consent was obtained from all participants. All research was performed in accordance with relevant guidelines/regulations, and in accordance with the Declaration of Helsinki.

### Experimental design

In this study, body-related information (overweight or underweight) was presented to participants through images of figure silhouettes (no racial distinction) prior to a food image in a food dot probe task, which is for activating the body self-schema. An image of a vase was presented to examine the baseline (neutral condition, no body-related information) status of women with BWD^38^. Participants were asked to complete the task, and eye tracking data (first fixation duration, direction) was recorded to investigate the pattern of food attentional bias.

### Questionnaires

#### Negative physical self scale-fatness (NPSS-F)

The negative physical self scale-fatness (NPSS-F) is a subscale of the negative physical self scale (NPSS). This 11-item subscale assesses body weight dissatisfaction among Chinese adolescents and adults. Items on the NPSS-F (e.g., “I am quite concerned about my weight” and “I am very distressed when I think about my weight”) are presented as statements related to body weight and are measured on a 5-point Likert scale ranging from 0 (*never*) to 4 (*always*). The average score is the scores of all the items (0–44) was summed up and divided by the number of items. Higher scores indicate greater dissatisfaction with body weight^1^. The NPSS-F had internally consistent scores (*α* = 0.88) as well as stable scores over 36 weeks (*r* = 0.70) among female students attending Chinese middle schools and high schools^39^. The reliability and validity of the NPSS-F has also been verified among North American women^40^. Cronbach’s α for this study was 0.92.

#### Hunger

Prior to the experiment, participants were asked to report their current level of hunger, ranging from 0 (*extremely hungry*) to 100 (*not hungry at all*).

#### Body mass index

Participants’ height and weight were measured and recorded to calculate their body mass index (BMI), which represents their body weight in kilograms divided by the square of their height in meters (kg/m^2^)^41^. This study followed the cutoff for BMI scores: a score below 18.5 is considered underweight, 18.5–24.9 normal weight, 25.0–29.9 overweight, and greater than or equal to 30 obese^41^.

### Stimuli

Food images in the experiment were obtained from the experimental food images library^42^. Five high-calorie food images (image numbers: 0018, 0026, 0169, 0286, 0351; kcal/100 g: *M* = 462.80 kcal, *SD* = 84.21), five low-calorie food images (image numbers: 0394, 0466, 0753, 0763, 0798; kcal/100 g: *M* = 58.00 kcal, *SD* = 21.54), and 20 neutral images (tools or instruments, image numbers: e.g., 1010, 1039, 1094, 1146, 1214) were selected as experimental materials (10 as filler materials). To control the influence of the edible degree of food, all food images were directly edible foods. All images were presented at a 15.6° × 11.7° viewing angle.

The stimuli images were divided into 15 pairs, and the food–neutral (F–N) pairs consisted of a food image and a neutral image, each with a food image on the left or a food image on the right in two cases, for a total of 10 pairs. The neutral–neutral (N–N) pairs consisted of two neutral images, for a total of 5 pairs. All paired images were matched as closely as possible on color and shape (paired images did not differ in color: *p* = 0.58 (red), *p* = 0.73 (green),* p* = 0.39 (blue); contrast, *p* = 0.23, brightness, *p* = 0.27, complexity, *p* = 0.51).

In body-related information from the Greenleaf et al.^38^ study on body contours (figure silhouettes), body contours were arranged as follows: very thin to obese into nine charts based on the definitions and scores of female body contours. To avoid selecting extreme images leading to participants recognizing the body contour map images as pathological body shapes, we selected two underweight and two overweight body contours, represented by Greenleaf et al.’s image numbers 2, 3, 7, and 8. All images were presented at a 11.8° × 14.8° viewing angle.

### Eye movement measurements

EyeLink® 1000 Plus (SR Research, Mississauga, Ontario, Canada) was used to record the eye tracking indicators. The experiment tracked the right eye at a sampling rate of 1000 Hz (the accurate calibration in the current experiment was better than 0.5° visual angle). Stimuli were presented with a white background on a 24-in screen at a resolution of 1280 × 960 pixels. The indices of eye tracking data were direction bias and first fixation duration bias of high-calorie foods and low-calorie foods. Direction bias reflects early attention distribution, and first fixation duration bias reflects the continuity of early attention distribution^37^. Standard velocity-based thresholds of the SR Research Experiment Builder software were used for event detection (velocity threshold for saccade detection: 30°/s). The effective fixation screening criteria were as follows^3, 27^: Fixations to a position were defined by an absence of saccades (saccades exceed within a 1° visual angle) and blinks for at least 100 ms^43^.

### Attentional bias scores

The response latency score was calculated by subtracting the mean response latency when the dot was consistent with the food image from when the dot was inconsistent with the food image. A positive score represented food attentional bias and a negative score indicated attentional avoidance^44^.

The direction bias score was a percentage score indicating the proportion of trials in which the first fixation fell on the food image to all trials in which the first fixations fell within the area of interest (15.6° × 11.7° viewing angle of food images and non-food images). Direction bias scores of more than 50% indicated early attention to food, and scores of less than 50% represented early food attentional avoidance. The first fixation duration bias score was calculated by subtracting the mean first fixation duration time in the non-food image from the mean first fixation duration time in the food image^35^. A positive score represented food attentional bias and a negative score indicated attentional avoidance^45^.

### Procedure

Participants were asked to refrain from eating any food 60 min prior to the experiments before they were invited to enter the eye tracking laboratory^46^, where they reported their current hunger, signed informed consent forms, and then completed the food dot probe task. The task lasted approximately 30–40 min. After the task, participants reported demographic information, and their height and weight were measured by the research assistant.

#### Dot probe task

**Figure 1:**
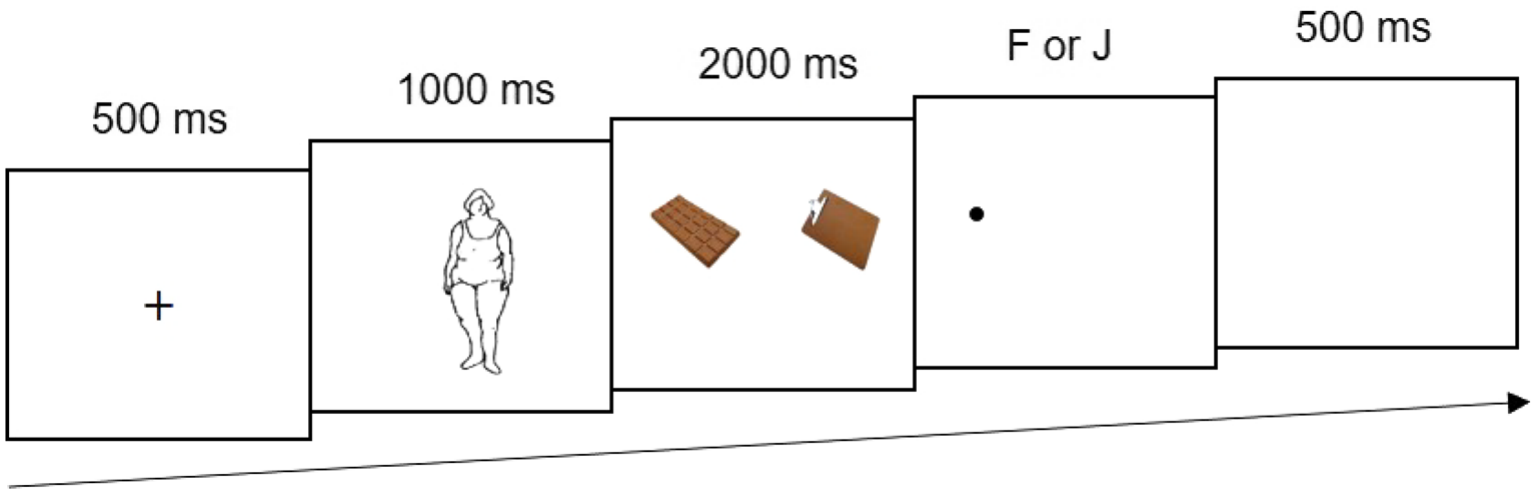
An example of a congruent critical trial in the dot probe task. In each trial, after a pair of food images disappeared, a black dot appeared randomly on the location of one of the two food images of the screen, and participants needed to press “F” or “J” to identify the location of the dot.

Participants were invited to the laboratory for the task (Fig. 1). The participants were seated 60 cm from the monitor screen, and a chin rest fixed head position. Before the dot probe task, participants completed a 9-dot calibration for eye movements. In the task, an image with body information appeared 22 times, the vase appeared 42 times, each F–N pair appeared 12 times, and each N–N pair appeared 2 times, for a total of 130 trials (120 experiment trials with 10 filler trials). A practice session was conducted to familiarize participants with the task, which included 15 trials. The sets of paired images in the practice session were different from those in the formal task. Each trial began with the presentation of a black central fixation cross “+” for 500 ms, followed by a body-related image (overweight, thin, neutral) in the center of screen for 1000 ms, followed by a pair of food images for 2000 ms. A black dot appeared randomly on the left or right side of the screen following the food images. The dot disappeared when the participant made a choice and a blank screen was shown for 500 ms, followed by the next trial.

### Statistical analysis

Data from practice trials and N–N trials used to balance the experiment were excluded. The response latency results were preprocessed, trials with errors were removed (0.82% of data), and trials on which response latencies was below 1 *SD* (from each participant’s mean) or greater than 3 *SD* (from each participant’s mean) were removed (0.45% of data). The accuracy for all participants was > 90%, and all participants were retained. Eye tracking results were preprocessed, and participants whose fixation point was less than 80% in the areas of interest were excluded (one BWD and three NBWDs). All data were analyzed using IBM® SPSS® Statistics Version 23.0, and a 2 (Group: BWD, NBWD) × 2 (food type: high calorie, low calorie) × 3 (Body-related information: overweight, thin, neutral) repeated measures ANOVA with covariate (BMI) was conducted for response latency score, first fixation duration bias score, and direction bias score. BMI was significantly different between the two groups, in accordance with previous studies that have shown an association between food attentional bias and BMI^35^.

## Results

### Response latency score

A significant main effect of body-related information was observed: *F*(2, 64) = 3.15, *p* = 0.046, partial η^2^ = 0.046. There was no significant main effects of group:* F* (1, 65) = 0.07, *p* = 0.788 or food type: *F*(1, 65) = 0.19, *p* = 0.663. No significant interaction effect was observed between group and body-related information: *F*(2, 64) = 1.67, *p* = 0.192, food type and body-related information: *F*(2, 64) = 1.38, *p* = 0.259, or group and food type: *F*(1, 65) = 0.15, *p* = 0.698. There was also no significant effect interaction among body-related information, group, and food type: *F*(2, 64) = 2.43, *p* = 0.096 (Table 2). Cronbach’s α for response latency score was 0.553.

**Table 2:** Descriptive statistics among variables.

Variables			BWD		NBWD	
Body-related information	Food type		*M*	*SE*	*M*	*SE*
Overweight	High calories	Response latency	− 3.07	9.19	− 1.93	8.52
		First fixation duration bias	195.29	56.43	− 18.78	54.20
		Direction bias	0.59	0.03	0.53	0.03
	Low calories	Response latency	− 11.67	8.24	12.63	7.64
		First fixation duration bias	34.12	52.48	25.75	50.41
		Direction bias	0.50	0.03	0.53	0.03
Thin	High calories	Response latency	− 19.12	11.33	9.32	10.50
		First fixation duration bias	97.81	45.03	106.67	43.25
		Direction bias	0.57	0.02	0.55	0.02
	Low calories	Response latency	0.14	13.90	− 10.67	12.88
		First fixation duration bias	64.67	48.91	39.84	46.98
		Direction bias	0.48	0.03	0.59	0.03
Neutral	High calories	Response latency	12.71	12.00	− 20.42	11.12
		First fixation duration bias	31.10	51.42	113.84	49.39
		Direction bias	0.55	0.03	0.60	0.03
	Low calories	Response latency	− 1.95	9.53	− 1.07	8.83
		First fixation duration bias	89.98	47.16	11.38	45.30
		Direction bias	0.57	0.03	0.50	0.03

*M* mean, *SE* standard error.

### First fixation duration bias score

**Figure 2:**
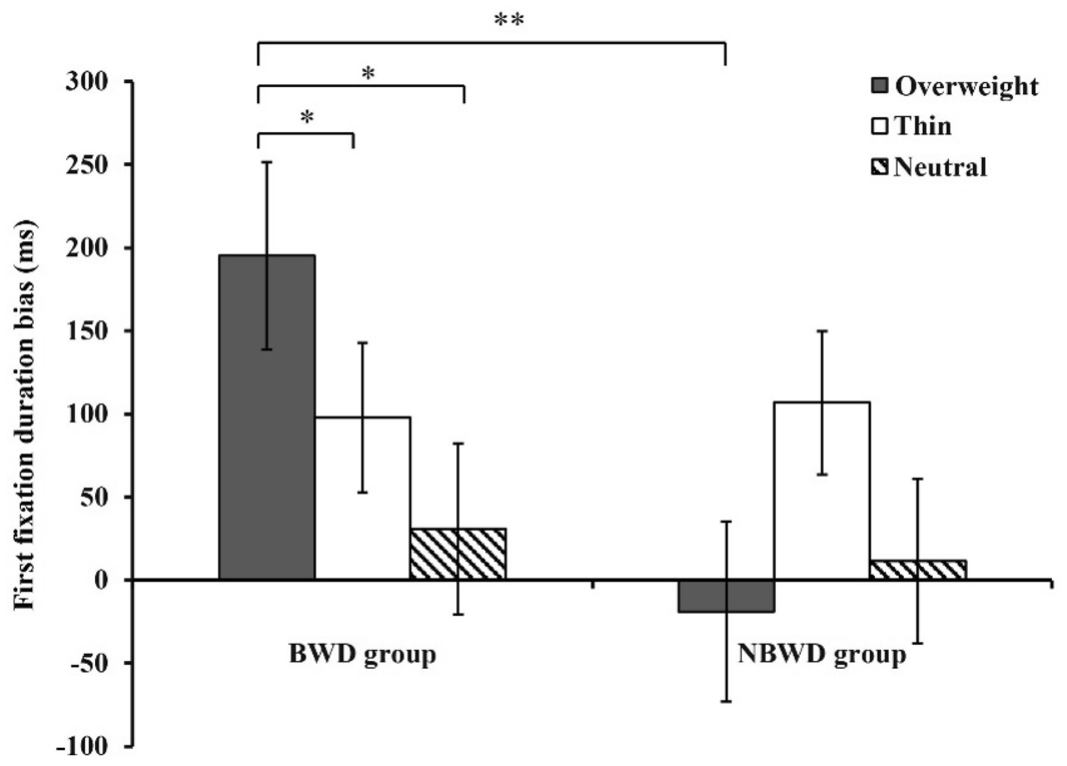
The comparison of first fixation duration bias score between women with body weight dissatisfaction and women with no body weight dissatisfaction on high-calorie food after exposure overweight/thin body-related information and neutral information. An asterisk (*) indicates *p* < 0.05, two asterisks (**) indicate *p* < 0.01. All *p* values are LSD corrected.

No significant main effects were observed for body-related information,* F*(2, 60) = 0.27, *p* = 0.761; group, *F*(1, 61) = 0.38, *p* = 0.541; or food type, *F*(1, 61) = 0.19, *p* = 0.664. There was no significant interaction between group and body-related information, *F* (2, 60) = 2.54, *p* = 0.087, food type and body-related information, *F*(2, 60) = 2.14, *p* = 0.122, or group and food type, *F*(1, 61) = 0.007, *p* = 0.933. However, there was a significant interaction between induced body-related information, group, and food type, *F*(2, 60) = 4.71, *p* = 0.013, partial η^2^ = 0.136 (Table 2). The simple effect analysis showed that, after exposure to thin body-related information and neutral information, there was no significant effect of group on food type(*p* = 0.288; *p* = 0.294;); however, after exposure to overweight cues, the main effect of group on high-calorie food was significant, *F*(1, 61) = 5.82, *p* = 0.019, partial η^2^ = 0.087; the BWD group had a higher first fixation duration bias score (*M* = 195.29, 95% CI [82.46, 308.12]) than the NBWD group (*M* = -18.78, 95% CI [-127.16, 89.60]). In the BWD group, there were significant main effects of body-related information on high-calorie foods, *F*(2, 60) = 4.47, *p* = 0.015, partial η^2^ = 0.130. An LSD paired *t* test was conducted, after exposure to overweight cues (*M* = 195.29, 95% CI [82.46, 308.12]), the first fixation duration bias score was higher than thin body-related information (*M* = 97.81, 95% CI [7.76, 187.85]), *t*(30) = 2.09, *p* = 0.048, Cohen’s* d* = 0.41, and neutral information (*M* = 31.10, 95% CI [-71.72, 133.92]), *t*(30) = 3.52, *p* = 0.004, Cohen’s* d* = 0.70 (Fig. 2). Cronbach’s α for first fixation duration bias score was 0.884.

### Direction bias score

**Figure 3:**
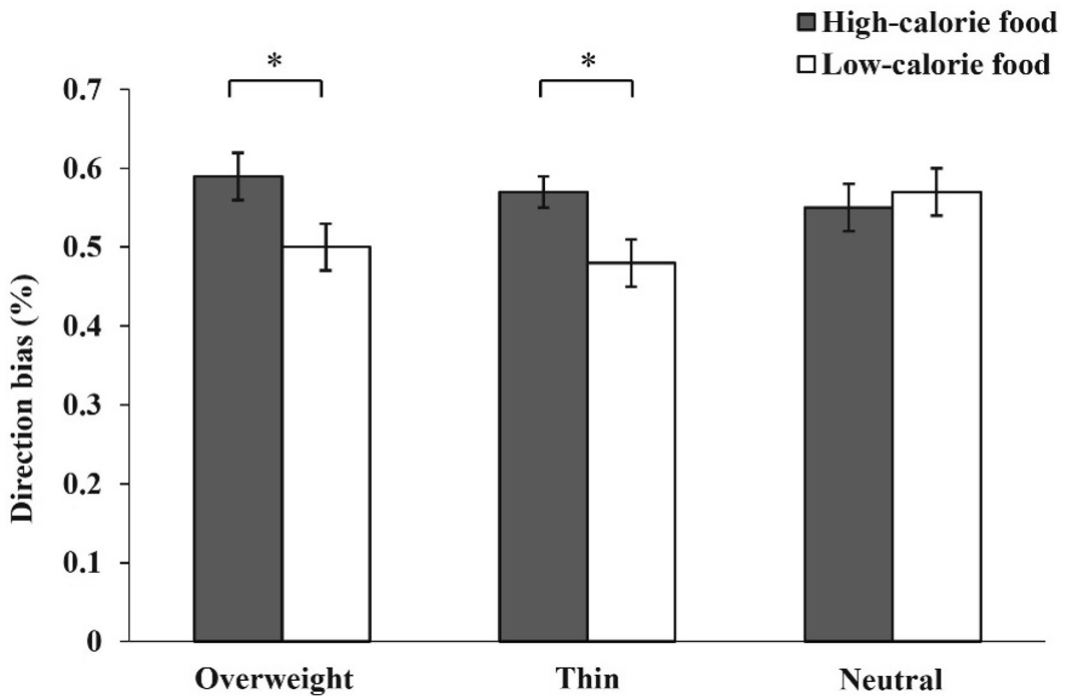
The comparison of the direction bias score between high-calorie food and low-calorie food in women with body weight dissatisfaction after exposure to overweight cues, thin body-related information, and neutral information. An asterisk (*) indicates *p* < 0.05. All *p* values are LSD corrected.

There were no significant main effects for body-related information, *F*(2, 60) = 1.49, *p* = 0.229; group,* F*(1, 61) = 0.07, *p* = 0.791; or food type, *F*(1, 61) = 0.28, *p* = 0.598. There was no significant interaction between group and body-related information: *F*(2, 60) = 2.18, *p* = 0.122, nor between food type and body-related information: *F*(2, 60) = 1.52, *p* = 0.224; or between group and food type: *F*(1, 61) = 0.80, *p* = 0.375. There was a significant interaction among body-related information, group, and food type: *F*(2, 60) = 3.75, *p* = 0.005, partial η^2^ = 0.111 (Table 2). The simple effect analysis showed that, in the BWD group, after exposure to neutral information, there was no significant main effect of food type (*p* = 0.295). However, after exposure to overweight cues, the main effect of food type in the BWD group was significant: *F*(1, 61) = 4.49, *p* = 0.038, partial η^2^ = 0.069, the direction bias score on high-calorie foods (*M* = 0.59, 95% CI [0.54, 0.65]) was more often than low-calorie foods (*M* = 0.50, 95% CI [0.44, 0.56]). After exposure to thin body-related information, the main effect of food type in BWD group was significant: *F*(1, 61) = 5.02, *p* = 0.029, partial η^2^ = 0.076, the direction bias score on high-calorie foods (*M* = 0.57, 95% CI [0.52, 0.62]) was more often than low-calorie foods (*M* = 0.48, 95% CI [0.42, 0.54]) (Fig. 3). In the NBWD group, there was no significant main effect of food type on body-related information (*p* = 0.305). Cronbach’s α for direction bias score was 0.330.

## Discussion

This study investigated the effect of body-related information on food attention using eye tracking technology. The baseline status (neutral condition) and thin-related condition, the BWD group, like the NBWD group, showed no difference in first fixation duration bias between high-calorie foods and low-calorie foods. However, the BWD group had more first fixation duration bias for high-calorie foods than for low-calorie foods and the NBWD group after exposure to overweight cues. Additionally, at baseline (neutral condition), the BWD group showed no difference in direction bias between high- and low-calorie foods. However, after exposure to body-related information, the BWD group showed earlier attentional bias for high-calorie foods than low-calorie foods.

The attentional bias of women with BWD supported our hypotheses. In quotidian life, ubiquitous body-related information may have an adverse effect on women with BWD. Mainstream media defines what a beautiful body is, and the standard is constantly changing^44^. Women receive an onslaught of body-related information from various sources, such as social media and fashion advertising. They often feel self-body dissatisfaction when they are exposed to overweight or underweight models, because they routinely compare themselves to these models^35^. For example, when women with BWD engage with overweight or underweight models, they may view themselves having the same body shape as these models^48^. This may lead to an undesirable process if they consider being overweight or underweight unacceptable or unattractive^49^—if women with BWD experience negative feelings after they contact overweight or underweight cues, they may assimilate their own bodies as unattractive, which could result in negative states^33^. This process is consistent with the cognitive-behavioral model—that is, body-related information easily activates negative cognitive biases in BWD^24^. A previous study found that negative affect may impair an individual's inhibitory control, thereby increasing the possibility of food intake^50^. The results of our study are consistent with this process.

To alleviate the negative self-evaluation induced by overweight cues, women with BWD tend to change their attention to food cues containing external reward information. Our results support the theory of food reward^20^, as high-calorie foods may be considered factors to alleviate negative moods. This attention process may be an unconscious decision as—many studies have posited that biased information processing occurs in an automatic fashion outside of an individual’s awareness^24^. It is difficult for individuals to separate from this process when they are fixated on high-calorie foods, and automatic attentional bias can trigger an increase in appetite^51, 52^. Our results were consistent with previous studies that found that comparing themselves to overweight images was a negative process that may lead to unhealthy eating behaviors in women with BWD.

Conversely, a previous study found that exposure to overweight models had a positive effect on women^33^. However, there are certain differences when compared with our study. That study used self-reports to assess exposure effects and their study participants were female undergraduate students^33^, whereas our study used eye tracking to record the effect of body exposure in food cue processing and our study participants were women with BWD, who had a specific attitude toward body-related information. Women with BWD often have negative self-schema when they come into contact with body-related information that may tend to lead to self-defeating behaviors. This is consistent with Dalley et al.’s^53^ study’s results, which found that women are vulnerable to body image dissatisfaction upon exposure to body media images, and that BMI and neuroticism exacerbates their body image dissatisfaction after exposure to overweight images.

Interestingly, we found an increased initial orientation (direction) toward high-calorie foods in women with BWD after exposure to thin body-related information. In general, women without BWD tend to compare themselves to thin body images (upward comparisons), and are more likely to show increased body dissatisfaction^54^. Women with BWD may also be affected by this process. Another possibility is that thin body images induce the incentive of women with BWD to lose weight, and their early attentional bias may be an implicit cognitive strategy of vigilantly screening threatening food cues in their surrounding environment to resist potential food temptation to facilitate restrictive eating^24^. The lack of difference in response latency between women with BWD and women with NBWD may be due to the poor internal reliability of response latency, and the high internal reliability of first fixation duration—therefore, it is possible that response latencies were not reliable enough to assess any biases. This is consistent with the reliability of attentional bias for food in our study^32^. Our results showed that direction bias had poor internal and test–retest reliability, first fixation duration bias had excellent internal and acceptable test–retest reliability, and response latency had acceptable internal and test–retest reliability^32^.

The unhealthy diets of women with BWD are likely to be affected by body-related information in their surroundings^55^. Our study’s findings have important implications for preventing eating disorders. Beauty should be associated with body health—not body size or shape—and social media aimed at reducing the anxiety caused by obesity may decrease dissatisfaction and protect self-esteem in women with BWD. Additionally, clinical interventions should guide women with BWD toward developing a reasonable concept of their body and appearance to facilitate coping with the overweight cues of fashion advertisements and maintaining a healthy diet.

### Limitations

Although the eye tracking technology used in this study is relatively reliable for exploring food attention in women with BWD, neurobiological evidence is lacking. Food could be a reward for women with BWD to alleviate negative states caused by body-related information^56^. Meanwhile, liking and disliking food stimuli could trigger individuals to choose or avoid certain foods^57^. Further research should consider the effect of liking. Otherwise, high-calorie foods also can be viewed as threat stimuli, because they could disrupt individuals’ goals of keeping healthy and losing weight^58^, and this may affect the attention bias of food. Future studies could use neurobiological techniques (such as functional magnetic resonance imaging technology) to explore the cognitive process of food attention after exposure to body-related information in women with BWD. Future studies should also use longer food-cue exposure times and investigate if the attention biases found our study are also present in prolonged attention. Previous studies that adopted longer viewing times of food images (e.g. 6000 ms^59^, 2020; 8000 ms^60^, 2022) found a difference of attention bias between high-calorie foods and low-calorie foods. Future research should also focus on interventions for body weight dissatisfaction, such as attentional bias modification training to change food intake in women with BWD.

## Conclusion

Our study found that when exposed to overweight cues, women with BWD showed early attentional bias and longer first fixation duration of high-calorie foods, which indicates that they may be affected by body-related information. The cognitive process of this result is that overweight cues may activate the negative self-evaluation of women with BWD and change their attention to high-calorie foods. The present study identified possible cognitive processes of eating behaviors in women with BWD. Therefore, body-related information could be an important inducing factor of unhealthy eating behaviors in women, and the effect of body-related information on public health should be considered by researchers, healthcare professionals, policymakers, and other stakeholders (Supplementary Information S1).

### Supplementary Information

Supplementary Information 1.

Supplementary Information 2.

## Data Availability

The data that support the findings of this study are openly available in [Figshare] at https://doi.org/10.6084/m9.figshare.19535566.
