# Supplementary material for: Effect of body-related information on food attentional bias in women with body weight dissatisfaction
Source: Sci Rep. 2023 Oct 4;13:16736. doi: 10.1038/s41598-023-43455-6 (PMC10551023; doi:10.1038/s41598-023-43455-6)
Supplement: Supplementary file 1 — Supplementary Information 1. [file 41598_2023_43455_MOESM1_ESM.rtf]

[1] -- Friday, September 08, 2023 -- 18:06:16
F tests - ANOVA: Repeated measures, within-between interaction
Analysis:	A priori: Compute required sample size 
Input:	Effect size f	=	0.2
	á err prob	=	0.05
	Power (1-â err prob)	=	0.8
	Number of groups	=	2
	Number of measurements	=	3
	Corr among rep measures	=	0.5
	Nonsphericity correction å	=	1
Output:	Noncentrality parameter ë	=	10.0800000
	Critical F	=	3.1107662
	Numerator df	=	2.0000000
	Denominator df	=	80.0000000
	Total sample size	=	42
	Actual power	=	0.8031391
