# Supplementary material for: Effect of body-related information on food attentional bias in women with body weight dissatisfaction
Source: Sci Rep. 2023 Oct 4;13:16736. doi: 10.1038/s41598-023-43455-6 (PMC10551023; doi:10.1038/s41598-023-43455-6)
Supplement: Supplementary file 2 — Supplementary Information 2. [file 41598_2023_43455_MOESM2_ESM.docx]

**Effect of Body-Related Information on Food Attentional Bias in Women with Body Weight Dissatisfaction**

***Attentional bias scores***

The dwell time bias score was calculated by subtracting the mean total dwell time in the non-food image from the mean total dwell time in the food image. A positive score represented food attentional bias and a negative score indicated attentional avoidance [1].

***Statistical analysis***

All data were analyzed using IBM® SPSS® Statistics Version 23.0, and a 2 (Group: BWD, NBWD) × 2 (food type: high calorie, low calorie) × 3 (Body-related information: overweight, thin, neutral) repeated measures ANOVA with covariate (BMI) was conducted for dwell time. BMI was significantly different between the two groups, in accordance with previous studies that have shown an association between food attentional bias and BMI [2].

**Results**

***Dwell time bias***

No significant main effects were observed for body-related information, *F*(2, 60) = .48, *p* = .620; group, *F*(1, 61) = 1.34, *p* = .251; or food type, *F*(1, 61) = .06, *p* = .807. There was no significant interaction between group and body-related information, *F* (2, 60) = .32, *p* = .729, food type and body-related information, *F*(2, 60) = 0.29, *p* = .751, or group and food type, *F*(1, 61) = .072, *p* = .398. There was a marginal significant interaction between induced body-related information, group, and food type, *F*(2, 60) = 3.05, *p* = .055, partial η^2^ = .092. The simple effect analysis showed that, after exposure to overweight cues, the main effect of group on high-calorie food was significant, *F*(1, 61) = 5.09, *p* = .019, partial η^2^ = .077; the BWD group had a higher dwell time bias score (*M* = 91.15, 95% CI [45.27, 137.04]) than the NBWD group (*M* = 11.72, 95% CI [-34.44, 55.89]). In the BWD group, there were significant main effects of high-calorie foods on body-related information, *F*(1, 61) =10.61, *p* = .002, partial η^2^ = .143. An LSD paired *t* test was conducted, the dwell time bias score was higher on high-calorie foods(*M* = 91.15, 95% CI [45.27, 137.04]) than low-calorie foods(*M* = 12.44, 95% CI [-27.72, 52.61]) after exposure to overweight cues *t*(30) = 2.64, *p* = 0.014, Cohen’s *d* = 0.47, and after exposure to thin cues *t*(30) = 3.03 *p* = 0.005, Cohen’s *d* = 0.54.

| **Table**  Descriptive statistics among Dwell time. | | | | | |
| --- | --- | --- | --- | --- | --- |
| Variables | | BWD | | NBWD | |
| Body-related information | food type | *M* | *SE* | *M* | *SE* |
| Overweight | high calories | 91.15 | 22.95 | 11.72 | 22.09 |
|  | low calories | 12.45 | 20.09 | 21.13 | 19.33 |
| Thin | high calories | 81.09 | 20.93 | 64.65 | 20.14 |
|  | low calories | 15.58 | 21.51 | 3.57 | 20.70 |
| Neutral | high calories | 46.39 | 22.02 | 40.87 | 21.20 |
|  | low calories | 38.23 | 17.32 | -1.69 | 16.67 |

The result of dwell time bias is consistent with First fixation duration bias.

**References**

1. Castellanos, E. H. *et al.* Obese adults have visual attention bias for food cue images: evidence for altered reward system function. *Int. J. Obes.* **33**, 1063-1073 (2009).

2. Gao, X., Wang, Q. C., Chen, H., Wang, B. Y. & Zhao, G. Time course of attentional bias components toward body-shape related pictures among women with fat negative physical self: an eye movement study. *Acta Psychol.Sin.* **44**, 498-510 (2012).
